# Inhibition of the Ubiquitin-Activating Enzyme UBA1 Suppresses Diet-Induced Atherosclerosis in Apolipoprotein E-Knockout Mice

**DOI:** 10.1155/2020/7812709

**Published:** 2020-03-20

**Authors:** Jiawei Liao, Xiaolei Yang, Qiuyue Lin, Shuang Liu, Yunpeng Xie, Yunlong Xia, Hui-Hua Li

**Affiliations:** ^1^Department of Cardiology, Institute of Cardiovascular Diseases, First Affiliated Hospital of Dalian Medical University, Dalian 116011, China; ^2^Department of Occupational and Environmental Health, School of Public Health, Dalian Medical University, Dalian 116011, China

## Abstract

**Background:**

Ubiquitin-like modifier activating enzyme 1 (UBA1) is the first and major E1 activating enzyme in ubiquitin activation, the initial step of the ubiquitin-proteasome system. Defects in the expression or activity of UBA1 correlate with several neurodegenerative and cardiovascular disorders. However, whether UBA1 contributes to atherosclerosis is not defined.

**Methods and Results:**

Atherosclerosis was induced in apolipoprotein E-knockout (*Apoe-/-*) mice fed on an atherogenic diet. UBA1 expression, detected by immunohistochemical staining, was found to be significantly increased in the atherosclerotic plaques, which confirmed to be mainly derived from lesional CD68^+^ macrophages via immunofluorescence costaining. Inactivation of UBA1 by the specific inhibitor PYR-41 did not alter the main metabolic parameters during atherogenic diet feeding but suppressed atherosclerosis development with less macrophage infiltration and plaque necrosis. PYR-41 did not alter circulating immune cells determined by flow cytometry but significantly reduced aortic mRNA levels of cytokines related to monocyte recruitment (*Mcp-1*, *Vcam-1*, and *Icam-1*) and macrophage proinflammatory responses (*Il-1β* and *Il-6*). Besides, PYR-41 also suppressed aortic mRNA expression of NADPH oxidase (*Nox1*, *Nox2*, and *Nox4*) and lesional oxidative stress levels, determined by DHE staining. *In vitro*, PYR-41 blunted ox-LDL-induced lipid deposition and expression of proinflammatory cytokines (*Il-1β* and *Il-6*) and NADPH oxidases (*Nox1*, *Nox2*, and *Nox4*) in cultured RAW264.7 macrophages.

**Conclusions:**

We demonstrated that UBA1 expression was upregulated and mainly derived from macrophages in the atherosclerotic plaques and inactivation of UBA1 by PYR-41 suppressed atherosclerosis development probably through inhibiting macrophage proinflammatory response and oxidative stress. Our data suggested that UBA1 might be explored as a potential pharmaceutical target against atherosclerosis.

## 1. Introduction

Cardiovascular diseases have now ranked as the top cause of mortality globally, representing 31% of all global deaths in 2016, according to WHO's statistics [1]. Atherosclerosis, a chronic inflammatory disease with abnormal lipid deposition in the large- and median-sized arteries, is the predominant underlying contributor to the majority of fatal conditions, such as myocardial infarction, unstable angina, sudden cardiac death, and stroke [2]. Besides the strong inflammatory nature, atherosclerosis is also characterized by proliferation, apoptosis/necrosis, enhanced oxidative stress, and disturbed protein homeostasis [3].

The ubiquitin-proteasome system (UPS) is the major pathway for intracellular protein degradation within eukaryotic cells, accounting for 80-90% of intracellular protein degradation [4, 5]. Due to the central role of the UPS in maintaining intracellular protein metabolism, it is reported to be involved in a wide variety of biological processes, such as growth and proliferation, inflammation, oxidative stress, and apoptosis/necrosis, that are crucial for cardiovascular biology and pathology [6, 7]. The UPS includes two independent but sequential parts: the ubiquitination and proteasome-mediated proteolysis [4, 5]. As the initial step of the UPS, ubiquitination modification starts as ubiquitin activation by forming a thioester linkage with the E1 activating enzyme. Activated ubiquitin was then transferred through a transesterification reaction to an E2 conjugating enzyme, followed by formation of an isopeptide bond with targeted protein substrate via the E3 ligating enzyme [4, 5]. The ubiquitined proteins finally enter the 20S proteasome complex for further degradation into peptides [4, 5]. Previous studies have confirmed abnormal UPS in atherosclerosis, especially in symptomatic plaques, suggesting that the UPS contributes to atherogenesis [8–10]. However, current studies mainly focus on the proteasome and E3 ligating enzyme activities; little is known about the E1 activating enzymes, the apex of the ubiquitination cascade, in atherosclerosis.

Ubiquitin-like modifier activating enzyme 1 (UBA1) is the first identified E1 activating enzyme for ubiquitin activation [11]. It is a highly conserved protein and consists of two main isoforms: UBA1a containing 1058 amino acids and UBA1b containing 1018 amino acids [11]. Besides UBA1, UBA6 is also capable of activating ubiquitin [12]. However, UBA6-mediated ubiquitin activation is currently restricted to specific protein ubiquitination, as only one specific E2 enzyme out of the currently known E2 enzymes can be conjugated with UBA6 [12]. Therefore, UBA1 plays a central role in ubiquitin activation and protein quality. Total loss of UBA1 is lethal, while defects in UBA1 expression or activity contribute to the pathogenesis of several neurodegenerative disorders such as spinal muscular atrophy, Huntington's disease, Parkinson's disease, Alzheimer's disease, and amyotrophic lateral sclerosis [13]. Recent studies also suggested that UBA1 might be essential for maintaining cardiovascular homeostasis and thus contribute to disease progression. For example, Qin et al. reported that inhibition of UBA1 by a small molecular inhibitor PYR-41 effectively attenuated carotid neointimal thickening via suppressing monocyte/macrophage infiltration and smooth muscle cell proliferation in a rat model of balloon injury [14]; Shu et al. demonstrated that cardiac UBA1 was upregulated in a mouse model of angiotensin II-induced cardiac remodeling and UBA1 inhibition by PYR-41 significantly reduced cardiac inflammation and oxidative stress [15]. However, whether UBA1 is involved in atherosclerosis is not defined. Here in this study, we explored this issue in a mouse atherosclerosis model, the apolipoprotein E-knockout mice (*Apoe-/-*) fed on an atherogenic diet.

## 2. Materials and Methods

### 2.1. Animals and Experimental Designs

Male *Apoe-/-* mice on C57BL/6 background were purchased from Beijing Vital River Laboratory and housed under specific pathogen-free conditions on a 12-hour light/12-hour dark cycle with free access to water and diet. At the age of 10 weeks old, the mice were fed on an atherogenic diet (0.5% cholesterol and 20% fat) for the next 8 weeks to fast induce atherogenesis. PYR-41(Selleck) was administrated (10 mg/kg body weight) 2 times per week by intraperitoneal injection, with DMSO as control, during atherogenic diet feeding. All experiments were performed according to the guidelines for the care and use of laboratory animals of the National Institute of Health (NIH publication no.85Y23, revised 1996) and approved by the Animal Care and Use Committee of Dalian Medical University.

### 2.2. Plasma Lipid and Glucose Analysis

Mice were fasted for 4 hours. Blood samples were collected via retroorbital puncture. Plasma total cholesterol, triglycerides, and glucose were measured with commercial kits (BioSino), according to the manufacturer's guidance. Lipoprotein profiles were fractioned by fast protein liquid chromatography (FPLC) and analyzed as we previously described [16]. Insulin resistance was measured by glucose tolerance test (GTT). Briefly, mice were fasted for 4 hours and then challenged with an intraperitoneal injection of glucose (2 g/kg body weight). Blood samples were collected before (time 0) and at 30, 60, and 120 minutes (time 30, 60, and 120, respectively) after glucose injection by retroorbital bleeding. Plasma glucose was measured with commercial kit as described above (BioSino).

### 2.3. Histological Analysis

Mice were sacrificed and flushed with PBS through the left ventricle. The aorta and the aortic root samples were prepared as previously described [17]. Briefly, the aorta was rid of the adventitia and cut open longitudinally under a dissecting microscope, while the heart was embedded in OCT (Sakura Finetek), snap frozen in liquid nitrogen, and cross-sectioned serially at 7 *μ*m thick throughout the aortic root. Atherosclerosis burden in the *en face* aortas and the aortic root sections was visualized by Oil-red O (Sigma) staining. UBA1 expression in the vessels of the aortic root sections was detected by immunohistochemical staining with anti-UBA1 antibody (ab34711, Abcam) or immunofluorescence costaining using anti-UBA1 (ab34711, Abcam) and anti-CD68 (MCA1957, Bio-Rad) antibodies. Macrophages and smooth muscle cells in the atherosclerotic plaques were visualized by immunohistochemical staining with anti-CD68 antibody (MCA1957, Bio-Rad) and anti-*α*-SMA antibody (ab5694, Abcam). Necrotic areas in the plaques were visualized by HE staining and designated as eosin-negative acellular areas. All quantifications were determined with ImageJ software.

### 2.4. Flow Cytometry Analysis

Blood was collected via retroorbital puncture and depleted of erythrocyte by lysing buffer (BD Biosciences). Then cell suspensions were treated with Fc block, washed, and stained with CD45 PerCP-Cy5.5, CD3 FITC, CD11b FITC, F4/80 BV421, and Gr-1 APC (BD Biosciences) accordingly. Circulating monocytes were defined as CD45^+^CD11b^+^F4/80^+^Gr-1^−^, neutrophils as CD45^+^CD11b^+^Gr-1^+^F4/80^−^, and T cells as CD45^+^CD3^+^. Events were acquired on a live gate on Fortessa flow cytometer (BD Biosciences).

### 2.5. RNA Isolation and Quantitative Real-Time PCR Analysis

Total RNA was extracted with TRIzol (Invitrogen) and reverse transcripted using a RT kit (MedChem Express). Quantitative real-time PCR was performed using SYBR Green PCR reagents (MedChem Express) and normalized to *Gapdh*. The primer sequences used in quantitative real-time PCR are described in Table 1.

### 2.6. Cell Culture

RAW264.7 cells were cultured in RPMI-1640 medium (Gibco) supplemented with 10% fetal bovine serum and 1% antibiotics (100 Units/ml penicillin and 100 *μ*g/ml streptomycin) and maintained in a humidified incubator at 37°C under 5% CO_2_. When cells were grown to 80% confluence, they were treated with PYR-41 (5 *μ*M, Selleck) for 2 hours, then washed and subjected to ox-LDL (50 *μ*g/ml, Unionbiol) treatment. After incubation with ox-LDL for 12 hours, cells were harvested for RNA extraction or fixed with 4% paraformaldehyde solution for Oil-red O staining.

### 2.7. Statistical Analysis

Data were presented as mean or mean ± SEM. Statistical significance was evaluated by Student's *t*-test using Prism software. *P* value < 0.05 was regarded as significant.

## 3. Results

### 3.1. UBA1 Expression Was Upregulated and Mainly Derived from Macrophages in the Diet-Induced Atherosclerosis in Apoe-/- Mice

**Figure 1:**
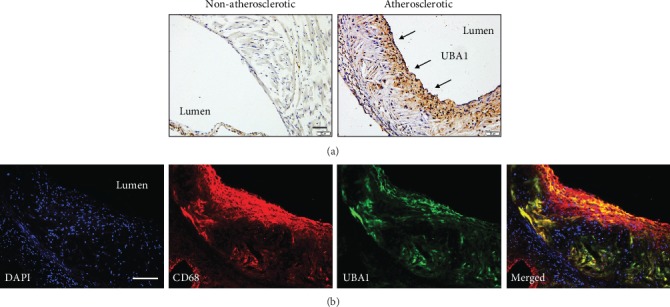
UBA1 expression was upregulated and mainly derived from macrophages in the atherosclerotic plaques of *Apoe-/-* mice. (a) Expression of UBA1 in the vessels with (right) or without atherosclerotic plaques (left). (b) Colocalization of UBA1 with the macrophage marker CD68 in the atherosclerotic plaques.

First, we explored the expression of UBA1 during atherogenesis in *Apoe-/-* mice. As shown in Figure 1(a), UBA1 expression was significantly increased in the diet-induced atherosclerotic plaques, detected by immunochemical analysis. To further identify the origin of UBA1 expression, we costained UBA1 with CD68, a marker of macrophages, which is the main type and also majority of functional cells in atherosclerosis. As shown in Figure 1(b), UBA1 almost fully colocalized with CD68. Together, our data indicated that UBA1 expression was upregulated and mainly derived from macrophage in diet-induced atherosclerosis in *Apoe-/-* mice.

### 3.2. Inhibition of UBA1 Did Not Alter the Main Metabolic Parameters during Diet-Induced Atherogenesis in Apoe-/- Mice

**Figure 2:**
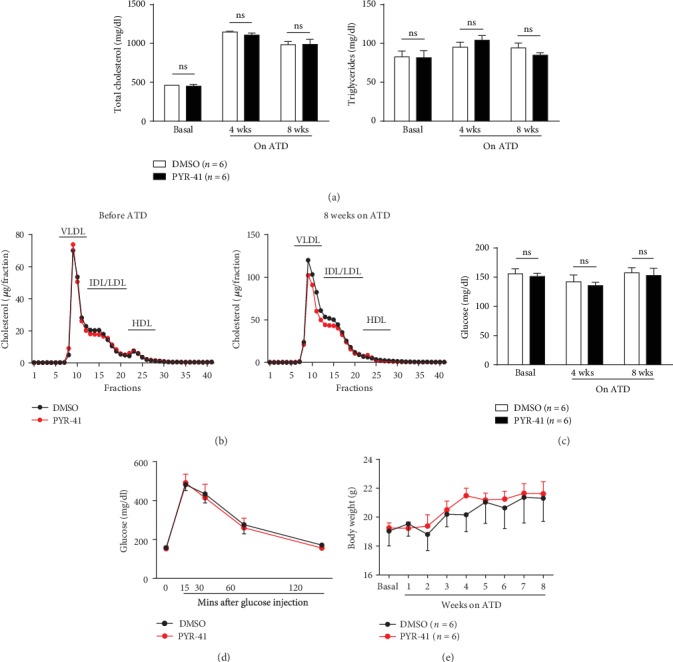
Inhibition of UBA1 did not alter the main metabolic parameters during diet-induced atherogenesis in *Apoe-/-* mice. (a) Plasma total cholesterol and triglyceride levels before and after the atherogenic diet feeding. (b) Plasma lipoprotein profiles fractioned by FPLC before (left) and after (right) the atherogenic diet feeding. (c) Plasma glucose levels before and after the atherogenic diet feeding. (d) Insulin resistance detected by the glucose tolerance test after 8 weeks on the atherogenic diet. (e) Body weight gain during the atherogenic diet feeding. *n* = 6 per group; ns: not significant; ATD: atherogenic diet.

Next, we examined the role of increased macrophage UBA1 expression in the diet-induced atherosclerosis in *Apoe-/-* mice, using a specific inhibitor PYR-41. We found that PYR-41 treatment did not change plasma total cholesterol (TC) and triglyceride (TG) levels as well as lipoprotein profiles fractioned by FPLC during atherogenic diet feeding (Figures 2(a) and 2(b)). Similarly, PYR-41 treatment did not change plasma glucose levels, insulin resistance determined by GTT, and body weight gain (Figures 2(c)–2(e)).

### 3.3. Inhibition of UBA1 Attenuated Diet-Induced Atherosclerosis in Apoe-/- Mice

**Figure 3:**
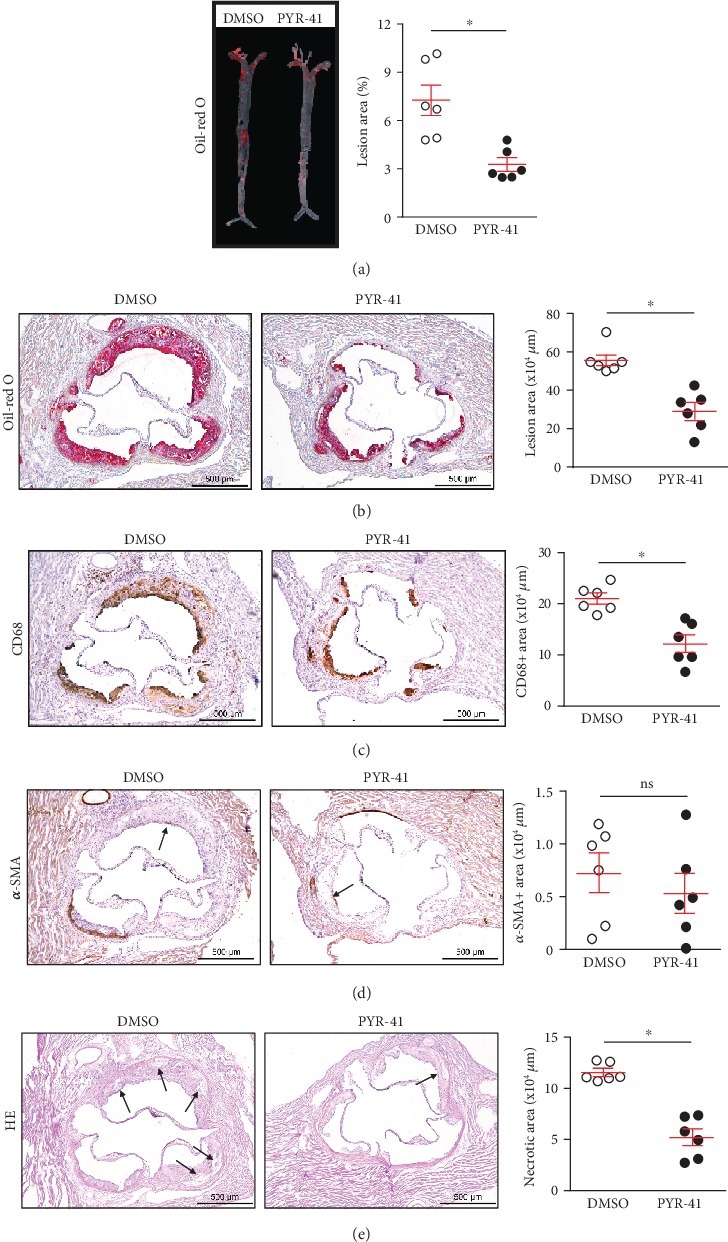
Inhibition of UBA1 attenuated diet-induced atherosclerosis in *Apoe-/-* mice. (a, b) Oil red O staining and quantitation of the atherosclerotic burden in the *en face* aorta (a) and the aortic root (b). (c) CD68 immunochemical staining and quantitation of the lesional macrophages in the aortic root. (d) *α*-SMA immunochemical staining and quantitation of the lesional smooth muscle cells in the aortic root. (e) HE staining and quantitation of the lesional necrotic areas in the aortic root. *n* = 6 per group; ∗: <0.05; ns: not significant.

We then explored the atherosclerosis development in the *Apoe-/-* mice with or without UBA1 inhibition by PYR-41, after 8 weeks on the atherogenic diet feeding. We found that PYR-41 treatment significantly reduced the atherosclerosis burden in the entire inner surface of the aortas and the aortic root, respectively (Figures 3(a) and 3(b)). Besides atherosclerosis burden, PYR-41 also changed the plaque composition, decreasing macrophage infiltration and plaque necrosis while not altering smooth muscle cell accumulation in the plaques (Figures 3(c)–3(e)). These data suggested that inhibition of UBA1 attenuated diet-induced atherosclerosis probably through inhibiting macrophage infiltration and plaque necrosis in *Apoe-/-* mice.

### 3.4. Inhibition of UBA1 Reduced Proinflammatory Cytokine Levels in Diet-Induced Atherosclerosis in Apoe-/- Mice

**Figure 4:**
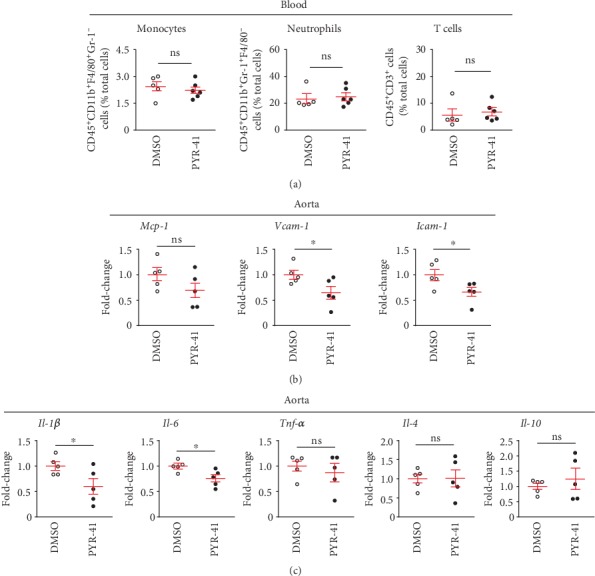
Inhibition of UBA1 decreased proinflammatory cytokine levels in diet-induced atherosclerosis in *Apoe-/-* mice. (a) Flow cytometry analysis of circulating monocytes, neutrophils, and T cells. (b) Quantitative real-time PCR analysis of aortic expression of cytokines associated with monocyte recruitment (*Mcp-1*, *Vcam-1*, and *Icam-1*). (c) Quantitative real-time PCR analysis of aortic expression of macrophage-derived proinflammatory (*Il-1β*, *Il-6*, and *Tnfα*) and anti-inflammatory (*Il-4* and *Il-10*) cytokines. *n* = 5‐6 per group; ∗: <0.05; ns: not significant.

We then explored whether UBA1 inhibition reduced circulating macrophages in diet-treated *Apoe-/-* mice. As shown in Figure 4(a), flow cytometry analysis revealed that PYR-41 treatment did not alter circulating monocytes, neutrophils, or T cells in the *Apoe-/-* mice received 2 weeks of atherogenic diet feeding. In the meantime, we observed that PYR-41 reduced the aortic expression of *Mcp-1*, *Vcam-1*, and *Icam-1*, key players in the monocyte recruitment, determined by real-time PCR analysis (Figure 4(b)). Furthermore, PYR-41 also inhibited the aortic expression of proinflammatory cytokines *Il-1β* and *Il-6* but did not alter the expression of anti-inflammatory *Il-4* or *Il-10* significantly (Figure 4(c)).

### 3.5. Inhibition of UBA1 Decreased Oxidative Stress in Diet-Induced Atherosclerosis in Apoe-/- Mice

**Figure 5:**
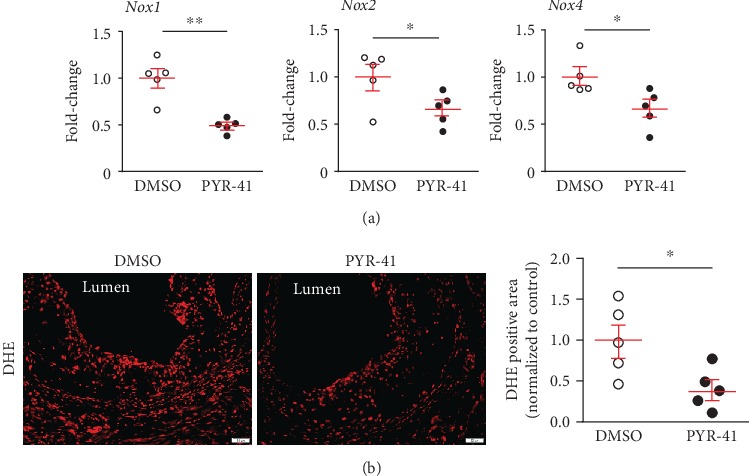
Inhibition of UBA1 decreased cellular oxidative stress in diet-induced atherosclerosis in *Apoe-/-* mice. (a) Quantitative real-time PCR analysis of aortic expression of NADPH oxidases (*Nox1*, *Nox2*, and *Nox4*). (b) DHE staining and quantitation of the lesional oxidative stress level in the aortic root. *n* = 5 per group; ∗: <0.05.

Previous studies have suggested that PYR-41 inhibited the NADPH oxidase (*Nox1*, *Nox2*, and *Nox4*) expression, leading to reduced cellular reactive oxygen species (ROS) generation and oxidative stress that could directly induce cell death [15]. Here, we confirmed that PYR-41 treatment decreased the aortic expression of *Nox1*, *Nox2*, and *Nox4* in the *Apoe-/-* mice fed on atherogenic diet for 2 weeks (Figure 5(a)). The reduced activity of *Noxs* further led to decreased ROS generation and oxidative stress in the atherosclerotic plaques, as shown by DHE staining, which might subsequently contribute to plaque necrosis (Figure 5(b)).

### 3.6. Inhibition of UBA1 Blunted Ox-LDL-Induced Lipid Accumulation and Expression of Proinflammatory Cytokines and NADPH Oxidases in Cultured Macrophages

**Figure 6:**
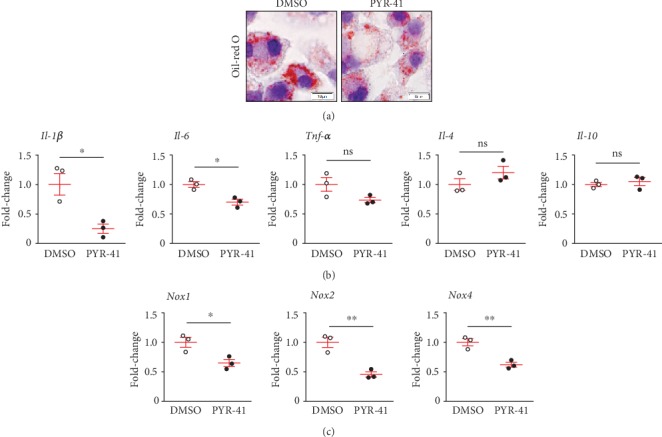
Inhibition of UBA1 blunted ox-LDL-induced lipid accumulation and expression of proinflammatory cytokines and NADPH oxidases in cultured macrophages. (a) Oil red O staining of ox-LDL-treated RAW264.7 cells. (b) Quantitative real-time PCR analysis of proinflammatory (*Il-1β*, *Il-6*, and *Tnfα*) and anti-inflammatory (*Il-4* and *Il-10*) cytokine expression in ox-LDL-treated RAW264.7 cells. (c) Quantitative real-time PCR analysis of NADPH oxidase (*Nox1*, *Nox2*, and *Nox4*) expression in ox-LDL-treated RAW264.7 cells. *n* = 3 per group; ∗: <0.05; ∗∗: <0.01; ns: not significant.

Finally, we explored whether PYR-41 could directly inhibit macrophage proinflammatory response and oxidative stress induced by oxidized low-density lipoprotein (ox-LDL) in a murine macrophage cell line, RAW264.7 cells. As shown in Figure 6(a), PYR-41 pretreatment significantly attenuated ox-LDL-induced lipid deposition in the RAW264.7 cells, shown by Oil-red O staining. Using real-time PCR analysis, we further showed that PYR-41 inhibited the expression of proinflammatory *Il-1β* and *Il-6* but did not alter the anti-inflammatory *Il-4* or *Il-10* expression (Figure 6(b)). PYR-41 also reduced the expression of *Nox1*, *Nox2*, and *Nox4* in ox-LDL-treated RAW264.7 cells (Figure 6(c)).

## 4. Discussion

In this study, we showed that UBA1 played an important role in the development of atherosclerosis in the *Apoe-/-* mice. The main findings of this study included (1) UBA1 expression was upregulated and mainly derived from macrophages in the diet-induced atherosclerotic plaques, (2) inhibition of UBA1 did not alter main metabolic parameters after atherogenic diet feeding, and (3) inhibition of UBA1 decreased atherosclerosis development by reducing macrophage inflammatory responses and oxidative stress. These results suggested that UBA1 inhibitor PYR-41 might represent a potential therapeutic agent for the treatment of atherosclerosis.

Atherosclerosis is primarily driven by the combination of arterial lipid deposition and inflammatory responses mediated by immune cells including monocyte-macrophages, neutrophils, and T cells [2]. Monocyte-derived macrophages play a crucial role in atherosclerosis. Upon recruitment by chemoattractant molecule *Mcp-1* and adhesion molecules *Vcam-1* and *Icam-1*, monocytes infiltrate the arterial walls and differentiate into macrophages, the latter uptake modified lipoproteins, in particular oxidized low-density lipoproteins, to form lipid-laden macrophages known as foam cells [18]. These foam cells can be typically classified as M1 cells toward proinflammatory cascade and plaque progression by secreting proinflammatory cytokines, such as *Il-1β*, *Il-6*, and *Tnfα*, and M2 cells toward inflammation and plaque regression by secreting anti-inflammatory cytokines, such as *Il-4* and *Il-10* [18, 19]. Besides cytokine secretion, foam cells also produce ROS, which are mainly mediated by NADPH oxidases (*Nox1*, *Nox2*, and *Nox4*), and therefore might cause oxidative stress that is capable of inducing cell death and necrotic core formation [20]. In this study, we observed that UBA1 expression was significantly increased and mainly derived from lesional macrophages in the atherosclerotic plaques. Further, using a small molecule inhibitor PYR-41, we demonstrated that UBA1 inactivation reduced macrophage proinflammatory responses, promoting macrophage phenotype turnover toward M1 cells. We also demonstrated that UBA1 inactivation suppressed oxidative stress by Nox-mediated ROS generation and therefore might be correlated with less macrophage death and necrotic core formation. Thus, our data for the first time identify a possible link between E1 activating enzyme and macrophage behaviors in atherosclerosis.

Currently, only a few E1/UBA1 inhibitors have been reported. PYR-41, whose full name is 4[4-(5-nitro-furan-2-ylmethylene)-3,5-dioxo-pyrazolidin-1-yl]-benzoic acid ethyl ester, is the first cell-permeable E1/UBA1 inhibitor, although the molecular basis is unknown [21]. Previous studies from our group have showed that PYR-41 is able to prevent the degradation of a wide range of target proteins, such as I*κ*B*α* and MKP-1 [15, 22]. Among them, I*κ*B*α* is an inhibitory protein for NF-*κ*B signaling pathway, which is known to play a critical role in regulating inflammatory cytokine and NADPH oxidase expression during cardiac remodeling and atherosclerosis [15, 23]. Interestingly, our recent data indicate that PYR-41 might suppress inflammation and oxidative stress possibly through stabilization of I*κ*B*α* and inhibition of NF-*κ*B activation in several cell and disease models [15, 22]. However, whether this is the case in atherosclerosis needs further exploration. In addition to inhibit NF-*κ*B signaling, PYR-41 also reduces p53 degradation and activates the transcriptional activity of this tumor suppressor, therefore might induce p53-expressing cell death [21]. Other E1/UBA1 inhibitors include chemical inhibitor NSC624206 [24] and some natural compounds such as panepophenanthrin, himeic acid A, largazole, and hyrtioreticulins A and B [25–28]. Studies of these inhibitors supported that UBA1 might be an attractive target for drug discovery to fight against cancer, neurodegenerative disorders, and infectious diseases [29, 30]. Our data for the first time identified an inhibitory effect of UBA1 inhibitor PYR-41 in atherosclerosis, therefore might expand the therapeutic potent of UBA1 as a potential pharmaceutical target against atherosclerosis.

## 5. Main Text

1. UBA1 expression was significantly increased and mainly derived from lesional macrophages in the diet-induced atherosclerotic plaques
2. Inhibition of UBA1 did not alter main metabolic parameters after atherogenic diet feeding
3. Inhibition of UBA1 suppressed atherosclerosis development by reducing macrophage proinflammatory responses and oxidative stress

## 6. Conclusions

In conclusion, we demonstrated that UBA1 expression was upregulated and mainly derived from macrophages in the atherosclerotic plaques of *Apoe-/-* mice. Inhibition of UBA1 decreased diet-induced atherosclerosis by reducing macrophage infiltration and plaque necrosis. Macrophage UBA1 might be explored as a potential pharmaceutical target against atherosclerosis.

## Figures and Tables

**Table 1:** Primer sequences used in the quantitative real-time PCR.

Name	Type	Sequence (5′-3′)
*Mcp-1*	Forward	TAAAAACCTGGATCGGAACCAAA
	Reverse	GCATTAGCTTCAGATTTACGGGT

*Vcam-1*	Forward	GTTCCAGCGAGGGTCTACC
	Reverse	AACTCTTGGCAAACATTAGGTGT

*Icam-1*	Forward	GCCTTGGTAGAGGTGACTGAG
	Reverse	GACCGGAGCTGAAAAGTTGTA

*Il-1β*	Forward	CTTCCCCAGGGCATGTTAAG
	Reverse	ACCCTGAGCGACCTGTCTTG

*Il-6*	Forward	TTCCATCCAGTTGCCTTCTTG
	Reverse	TTGGGAGTGGTATCCTCTGTGA

*Tnfα*	Forward	ATGGCCTCCCTCTCATCAGT
	Reverse	CTTGGTGGTTTGCTACGACG

*Il-4*	Forward	GGTCTCAACCCCCAGCTAGT
	Reverse	GCCGATGATCTCTCTCAAGTGAT

*Il-10*	Forward	GCTCTTACTGACTGGCATGAG
	Reverse	CGCAGCTCTAGGAGCATGTG

*Nox1*	Forward	CCCATCCAGTCTCCAAACATGAC
	Reverse	ACCAAAGCTACAGTGGCAATCAC

*Nox2*	Forward	CTTCTTGGGTCAGCACTGGC
	Reverse	GCAGCAAGATCAGCATGCAG

*Nox4*	Forward	CTTGGTGAATGCCCTCAACT
	Reverse	TTCTGGGATCCTCATTCTGG

*Gapdh*	Forward	AGGTCGGTGTGAACGGATTTG
	Reverse	GGGGTCGTTGATGGCAACA

## Data Availability

The data used to support the findings of this study are included within the article.

## Abbreviations

Apoe: Apolipoprotein E
*α*-SMA: *α*-Smooth muscle actin
ATD: Atherogenic diet
FPLC: Fast protein liquid chromatography
GTT: Glucose tolerance test
Icam-1: Intercellular adhesion molecule-1
I*κ*B: Inhibitor of nuclear factor-*κ*B
Il: Interleukin
Mcp-1: Monocyte chemoattractant protein-1
NF-*κ*B: Nuclear factor-*κ*B
Nox: NADPH oxidase
PYR-41: 4[4-(5-Nitro-furan-2-ylmethylene)-3,5-dioxo-pyrazolidin-1-yl]-benzoic acid ethyl ester
ROS: Reactive oxygen species
UBA1: Ubiquitin-like modifier activating enzyme 1
UPS: Ubiquitin-proteasome system
Vcam-1: Vascular cell adhesion molecule-1.
